# Association between serum creatinine and type 2 diabetes in the Chinese population: a retrospective cohort study

**DOI:** 10.1038/s41598-023-33878-6

**Published:** 2023-04-26

**Authors:** Rugang Li, Min He, Qilin Yang, Zezhi Liang, Ying li, Ling Huang, Rong Wu, Jieping Huang

**Affiliations:** 1 Department of Nephrology, Yuebei People’s Hospital, No. 133 South Huimin Road, Shaoguan, 512026 Guangdong China; 2 Department of Critical Care, the Second Affiliated Hospital of Guangzhou Medical University, No. 250 Changgang East Road, Haizhu District, Guangzhou, Guangdong China; 3 Department of Cardiology, Chenzhou Third People’s Hospital, Chenzhou, 423000 Hunan China

**Keywords:** Diseases, Endocrinology, Health care, Molecular medicine, Risk factors

## Abstract

The relationship between serum creatinine and type 2 diabetes is limited. We aimed to investigate the association of baseline serum creatinine and new-onset type 2 diabetes in Chinese population. This retrospective cohort study was conducted using data from the health screening program in China. The population were divided into four groups based on serum creatinine levels, and the outcome of interest was the occurrence of a diabetic event. Cox proportional risk model was used to assess the independent effect of baseline serum creatinine level on future diabetes risk. Sensitivity and subgroup analysis were used to verify the reliability of the results. After an average follow-up of 3.12 years, among 201,298 individuals aged ≥ 20 years, 3389 patients developed diabetes. Compared with participants in quartile 2–4 (> 51.6umol/L for female, > 71.8umol/L for male,), a significantly higher risk of new-onset Type 2 Diabetes (OR, 1.15; 95%CI: 1.07–1.23) was found in those in quartile 1 (< 51.6umol/L for female, < 71.8umol/L for male). Moreover, Similar results were found in various subgroups stratified by age, BMI, TG, TC, FPG and family history group. Low serum creatinine is independently associated with increased risk of type 2 diabetes in Chinese adults. It was also stable in various subgroups stratified.

## Introduction

The global prevalence of diabetes is estimated at 9.3% (463 million people) in 2019, possibly rising to 10.2% (578 million people) in 2030 and 10.9% (700 million people) in 2045^1^. As one the most common chronic diseases, diabetes has become an urgent public health problem worldwide. The escalating diabetes epidemic is being driven by a number of factors, including an aging population, economic development, urbanization, unhealthy eating habits and sedentary lifestyles^2^. It is also an important risk factor for atherosclerotic disease, which is a major cause of morbidity, mortality and expenditure in patients with diabetes^3^. Although care and complication rates improved prior to 2010^4^, complication rates have recently shown a worrying uptick, especially among young people^5^.Therefore, to fully understand the risk factors of diabetes is of great significance to the prevention and screening of diabetes.

Skeletal muscle is one of the main target organs of insulin and its material metabolism is regulated by insulin. Loss of skeletal muscle mass can lead to insulin resistance, which is a risk factor for type 2 diabetes^6,7^. Serum creatinine is primarily a metabolite of phosphocreatine in muscular tissue^8^. Because the creatine content per unit of skeletal muscle mass is consistent, as is the creatine breakdown rate, serum creatinine concentration is very stable and is a direct reflection of skeletal muscle mass^9–11^. In addition, imaging modes used to assess muscle mass, such as computed tomography, magnetic resonance imaging^12^ and ultrasound^13^,are often inconvenient and expensive, with radiation hazards. More recently, serum creatinine has been found to correlate closely with muscle mass as measured by magnetic resonance imaging^14,15^. Several cohort studies in single male workers have shown that low serum creatinine is a risk factor for type 2 diabetes^16–18^. Other cohort studies including men and women drew similar results^19–21^. But these studies were limited to the single-center or single-region. It still remains unclear whether these results will apply to a more general population.

To address this gap in knowledge, our current study aimed to assess the association between serum creatinine and new-onset type 2 diabetes and to examine possible effect modifiers in common population using data across China (N = 198,739, across 32 sites and 11 cities in China).

## Methods

### Data source

**Figure 1:**
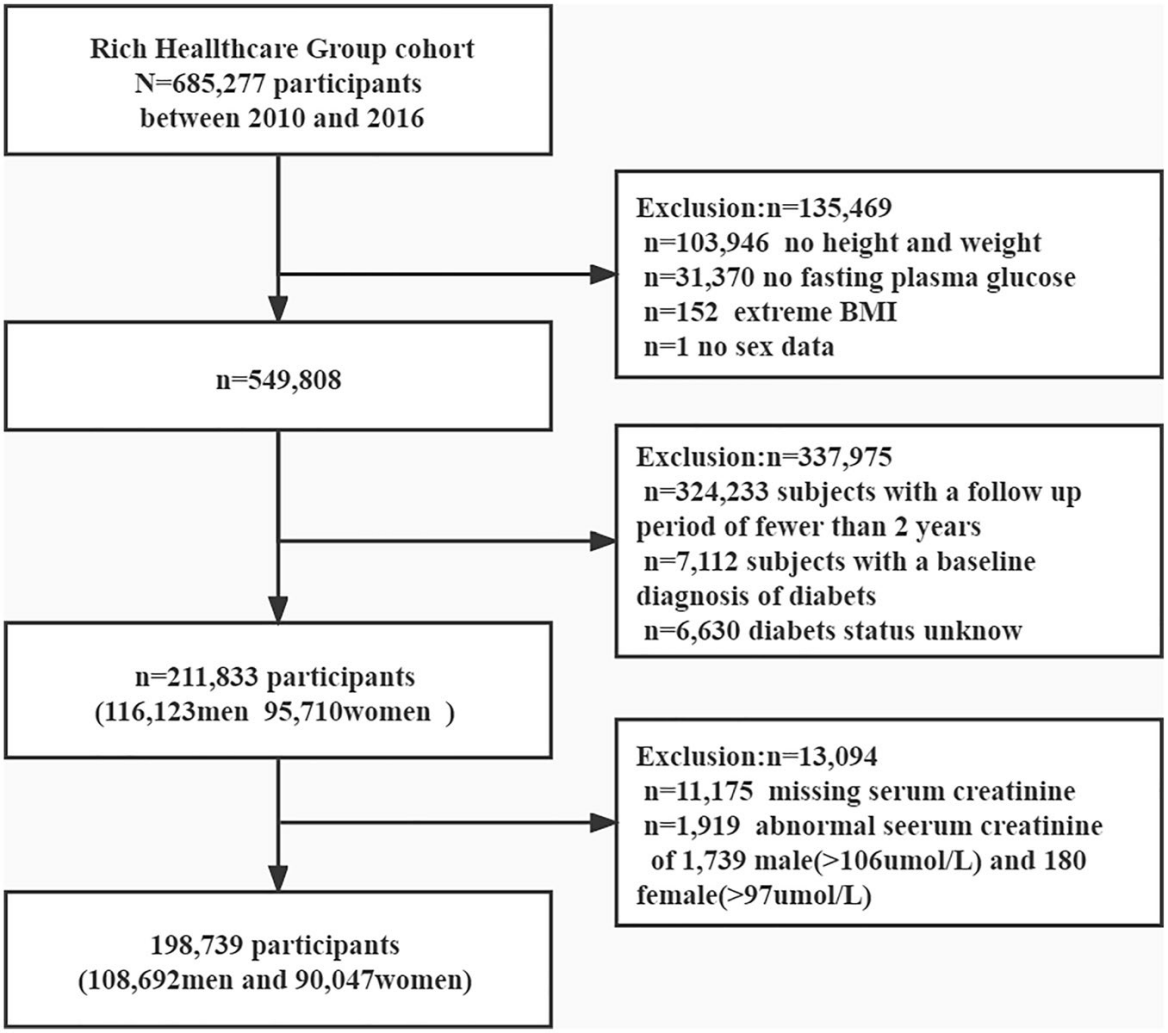
The flow chart of the study.

This study is a post-hoc analysis based on the design of a Chinese population cohort. The existing data have been collated by professor Chen and uploaded to the Andromeda database^22,23^.According to the Dryad database's terms of service, researchers are free to use public data for post-analysis in order to make the database more useful. Since the anonymity of the data and research ethics have been recognized in previous studies, there is no need to reapply for this study. In short, the Rich Health Care Group cohort study recruited 685,277 adult subjects older than 20 years of age from 11 cities in China including Beijing, Shanghai, Guangzhou, Shenzhen, Nanjing, Suzhou, Wuhan, Changzhou, Chengdu, Hefei, Nantong, who attended at least two health checkups between 2011 and 2016. The project aims to promote the health of the Chinese population and assess diabetes and its risk factors through health screening and follow-up of the general population. 211,833 subjects were included in the database after being sorted by the team professor Chen et al., as shown in Fig. 1. The exposure factor of this study was set to serum creatinine, which was of interest to the outcome of new diabetes events. We continued to exclude subjects with baseline creatinine deficiency (n = 11,175), and subjects with baseline creatinine greater than 106 umol/L in men (n = 1919) and 97 umol/L in women (n = 180) who may have renal insufficiency. A total of 198,739 participants were enrolled in the study.

### Data collection and measurements

Participants were asked to fill out a detailed questionnaire assessing demographics, lifestyle, medical history and family history of diabetes during each visit to the health screening center. Height, weight and blood pressure are measured by trained staff. The measurement of general physical signs requires the subject to wear light clothing and no shoes, with a weight reading of the nearest 0.1 kg and a height reading of the nearest 0.1 cm. BMI is calculated by dividing weight in kilograms by height in meters squared. Blood pressure was measured at rest with a standard mercury sphygmomanometer. Subjects were asked to fast for at least 10 h before each examination and then take fasting venous blood samples. Blood glucose was measured by glucose oxidase method on Beckman 5800 automatic analyzer. Serum creatinine was measured by enzyme on Beckman 5800 automatic analyzer. Serum triglyceride (TG), total cholesterol (TC), low density lipoprotein cholesterol (LDL-C) and high density lipoprotein cholesterol (HDL-C) were determined by Beckman 5800 automatic analyzer^22^.

### Study outcomes

The primary study outcome was new-onset type 2 diabetes, according to the American Diabetes Association criteria, defined as use of new onset FG ≥ 7.0 mmol/L, or self-reported during follow-up^24^. Subjects were censored by researcher at the date of diabetes diagnosis or at their last visit.

### Management of missing data

In the statistical analysis, covariables with missing values of continuous variables less than 6% were replaced by mean (normal distribution) and median (skewness distribution), and missing values greater than 6% were not included in the covariate analysis. Missing categorical variables are replaced with dummy variables.

### Statistical analysis

Patient characteristics were calculated according to sex-stratified serum creatinine quintiles. Data were expressed as mean and standard deviation (SD) when normally distributed or median and interquartile range (IQR) when skewed.

Baseline characteristics were presented as the mean ± SD for continuous variables or percentages for categorical variables. Differences in baseline characteristics by VAI tripartition were compared by chi-square test for categorical variables or analysis of variance (ANOVA) for continuous variables. The cumulative rates of new-onset type 2 diabetes were compared using the Kaplan–Meier curves and log-rank analyses.

The relationship of serum creatinine quartiles with new-onset type 2 diabetes was evaluated using Cox proportional hazards regression analyses models without or with adjustment of covariates including age, gender in Model I; and all the variables in Model I plus body mass index, systolic blood pressure, diastolic blood pressure, fasting plasma glucose, total cholesterol, triglyceride, blood urea nitrogen, alanine aminotransferase, drinking status, smoking status and family history of diabetes in Model II. Variables were selected as confounders based on existing literature and clinical judgment.

As additional exploratory analyses, possible modifications on the relationship of creatinine as a binary variable (< 71.8 vs. 71.8–106.0 umol/L for men; < 51.6 vs. 51.6–97.0 umol/L for women) with new-onset T2DM were assessed for variables including age (< 45 vs. ≥ 45 years), gender(male vs. female), BMI(< 21,21 ≤ 25 vs. ≥ 25 kg/m^2^), TG (< 1.7 vs. ≥ 1.7 mmol/L), TC (< 5.2 vs. ≥ 5.2 mmol/L), FPG (< 6.1 vs. ≥ 6.1 mmol/L), SBP (< 140 vs. ≥ 140 mmHg), DBP (< 90 vs. ≥ 90 mmHg), family history of diabetes (no vs. yes), smoking status (not recorded, current, former vs. non) and drinking status (not recorded, current, former vs. non) at baseline.

All the analyses were performed with the statistical software packages R 3.3.2 (http://www.R-project.org, The R Foundation) and Free Statistics software versions 1.3^25^. A two-tailed test was performed and *p *< 0.05 was considered statistically significant. We followed the Strengthening the Reporting of Observational Studies in Epidemiology (STROBE) reporting guideline.

## Results

### Study participants and baseline characteristics

A total of 198,739 participants (54.7% men and 45.3% women) were included in the analysis, the mean age of the population was 42.1 ± 12.6 years old. The mean year of follow up was 3.1 ± 0.9 years, and 3,930 people developed diabetes during follow-up. The mean creatinine was 69.6 ± 14.6 umol/L. The number of participants with missing data of SBP, DBP, and ALT, TC, TG, BUN, LDL-C and HDL were 20, 21, 1104, 3100, 3105, 11106, 82997 and 84008, respectively. Meanwhile, the missing data of smoking and drinking status were 141,681 and141,681.

Table1 depicted the baseline characteristics of the total population and by quartiles of the creatinine. We found that in highest creatinine group, participants generally had higher age, BMI, blood pressure levels (including both systolic and diastolic blood pressures), fasting blood glycemic, TC, LDL-C, creatinine and higher rates of current smoker and drinker. In contrast, there was no statistically significant difference in family history of diabetes and HDL-C among different creatinine groups.

**Table 1:** Baseline characteristics of participants.

Variables	Creatinine sex-stratified quartile(umol/L)				*P*-value
	Q1	Q2	Q3	Q4	
	F (< 51.6)	F (51.6 ≤ 57.0)	F (57.0 ≤ 63.0)	F (63.0–97.0)	
	M (< 71.8)	M (71.8 ≤ 79.0)	M (79.0 ≤ 86.6)	M (86.6–106.0)	
No of subjects	49381	48853	50049	50456	
Age,years	41.5 ± 11.9	41.4 ± 12.1	42.0 ± 12.5	43.6 ± 13.8	< 0.001
Gender, n (%)					< 0.001
male	27021 (54.7)	27092 (55.5)	27381 (54.7)	27198 (53.9)	
female	22360 (45.3)	21761 (44.5)	22668 (45.3)	23258 (46.1)	
Creatinine	57.5 ± 10.1	66.0 ± 10.6	72.2 ± 11.5	82.4 ± 13.1	< 0.001
male	65.9 ± 4.7	75.4 ± 2.0	82.5 ± 2.2	93.4 ± 5.1	< 0.001
female	47.3 ± 3.4	54.3 ± 1.5	59.7 ± 1.7	69.5 ± 6.0	< 0.001
BMI(kg/m^2^ )	23.1 ± 3.4	23.1 ± 3.3	23.2 ± 3.3	23.4 ± 3.3	< 0.001
SBP(mmHg)	119.2 ± 16.3	118.4 ± 16.0	118.6 ± 16.2	119.6 ± 16.8	< 0.001
DBP(mmHg)	74.2 ± 10.9	74.0 ± 10.7	74.0 ± 10.6	74.2 ± 10.9	< 0.001
FPG(mmol/L)	4.9 ± 0.6	4.9 ± 0.6	4.9 ± 0.6	4.9 ± 0.6	< 0.001
Family history of diabetes	1069 (2.2)	1046 (2.1)	1011 (2.0)	1038 (2.1)	0.334
TC(mmol/L)	4.7 ± 0.9	4.7 ± 0.9	4.7 ± 0.9	4.8 ± 0.9	< 0.001
TG(mmol/L)	1.3 ± 1.2	1.3 ± 1.0	1.3 ± 1.0	1.4 ± 1.0	< 0.001
LDL-C(mmol/L)	2.7 ± 0.7	2.8 ± 0.7	2.8 ± 0.7	2.8 ± 0.7	< 0.001
HDL-C(mmol/L)	1.4 ± 0.3	1.4 ± 0.3	1.4 ± 0.3	1.4 ± 0.3	0.463
BUN(mmol/L)	4.4 ± 1.1	4.6 ± 1.1	4.7 ± 1.1	4.9 ± 1.2	< 0.001
ALT(IU/L)	18.4 (13.0, 28.6)	18.0 (12.9, 27.8)	18.0 (12.9, 27.1)	18.0 (13.0, 26.8)	< 0.001
AST(IU/L)	22.0 (18.5, 27.0)	22.0 (18.5, 26.7)	22.0 (18.5, 26.1)	22.0 (18.9, 27.0)	< 0.001
Drinking status (%)					< 0.001
Not recorded	34771 (70.4)	34186 (70)	35583 (71.1)	37141 (73.6)	
Current	395 (0.8)	313 (0.6)	322 (0.6)	237 (0.5)	
Former	2040 (4.1)	2161 (4.4)	2204 (4.4)	2137 (4.2)	
Non	12175 (24.7)	12193 (25.0)	11940 (23.9)	10941 (21.7)	
Smoking status (%)					< 0.001
Not recorded	34771 (70.4)	34186 (70)	35583 (71.1)	37141 (73.6)	
Current	3142 (6.4)	2932 (6.0)	2746 (5.5)	2398 (4.8)	
Former	631 (1.3)	650 (1.3)	584 (1.2)	579 (1.1)	
Non	10837 (21.9)	11085 (22.7)	11136 (22.3)	10338 (20.5)	

*F* female, *M* male, *BMI* body mass index, *SBP* systolic blood pressure, *DBP* diastolic blood pressure, *FPG* fasting plasma glucose, *TC* total cholesterol, *TG* triglyceride, *LDL-C* low-density lipid cholesterol, *HDL-C* high-density lipoprotein cholesterol, *BUN* blood urea nitrogen, *ALT* alanine aminotransferase, *AST* aspartate aminotransferase.

### Association between creatinine and study outcomes

During a median of 3.1 years of follow-up (interquartile range 2.2–3.9), a total of 3,930 (2.0%) participants developed new-onset type 2 diabetes. Serum creatinine was lower in patients with new diabetes than in patients without new diabetes (57.5 ± 10.1vs. 82.4 ± 13.1 umol/L, *p *< 0.001).

**Figure 2:**
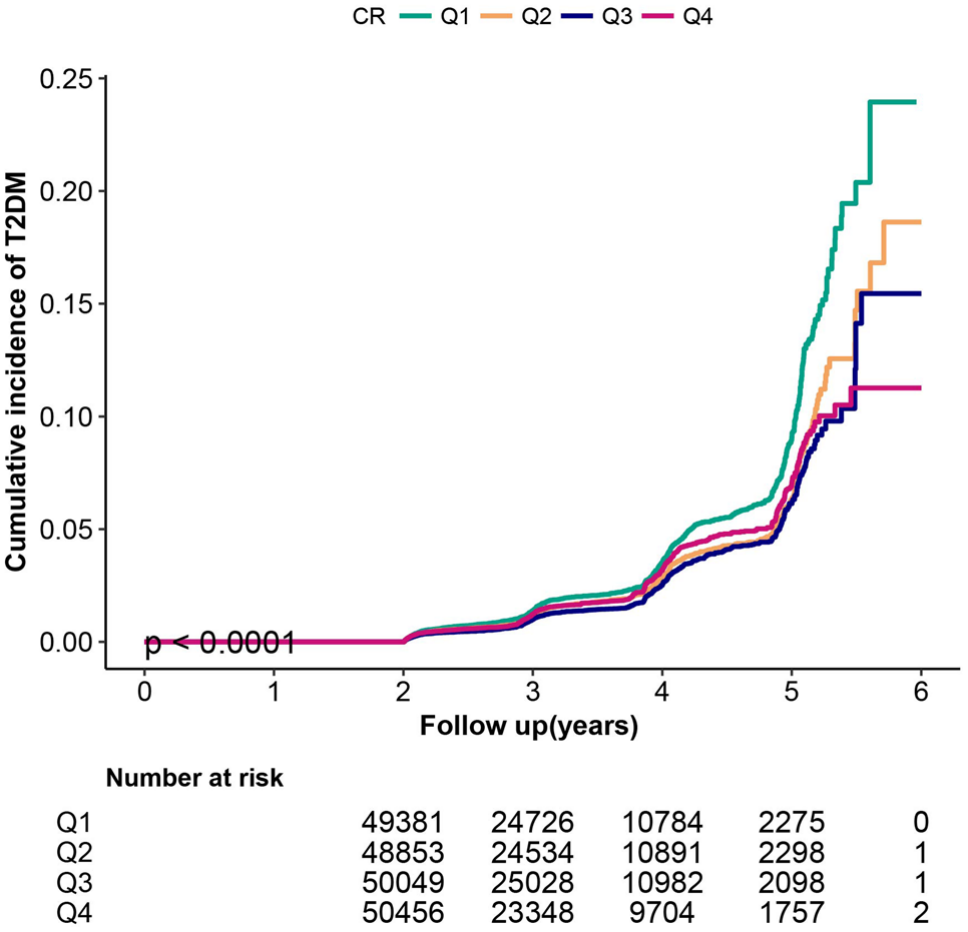
Kaplan–Meier event-free survival curve. Kaplan–Meier analysis of incident of diabetes based on creatinine quartiles (logrank, *P *< 0.0001).

Figure 2 shows the Kaplan–Meier curve for cumulative risk of diabetes event risk stratified by creatinine category. There were significant differences in the risk of developing diabetes among grade 4 groups (log-rank test, *p *< 0.0001). As creatinine decreased, the cumulative risk of diabetic events increased, presenting an array of the first four quartiles with the highest risk of diabetic events.

Table 2 shows the HRs and 95% CI for risk of diabetes as determined by serum creatinine levels. In the unadjusted model, the risk of diabetes increased with a one-quarter decrease in creatinine (P trend *p *< 0.0001). The lowest quartile of creatinine concentration was associated with a 1.47-fold increased risk of developing diabetes compared with the high quartile (adjusted HR, 1.47; 95% CI, 1.34–1.60).Adjusted hazard ratios of model 1(age, sex) and model 2(the variables in the model I, plus body mass index,systolic blood pressure,diastolic blood pressure,fasting plasma glucose,total cholesterol,triglyceride,blood urea nitrogen,alanine aminotransferase,drinking status,smoking status and family history of diabetes) were 1.52(1.40,1.66) and 1.21(1.11,1.33), respectively, (P trend *p *< 0.0001).

**Table 2:** Association between Creatinine and Type 2 Diabetes.

Creatinine	n.event(%)	Crude Model		Adjusted Model I		Adjusted Model II	
		HR (95%CI)	*P* value	HR (95%CI)	*P* value	HR (95%CI)	*P* value
*Creatinine Quartiles*							
Q1	1230(2.5)	1.47 (1.34–1.60)	< 0.001	1.52 (1.40–1.66)	< 0.001	1.21 (1.11–1.33)	< 0.001
Q2	940 (1.9)	1.13 (1.03–1.24)	0.013	1.16 (1.06–1.27)	0.002	1.09 (0.99–1.20)	0.069
Q3	837 (1.7)	Ref		Ref		Ref	
Q4	923 (1.8)	1.19 (1.09–1.31)	< 0.001	1.03 (0.94–1.13)	0.496	1.08 (0.98–1.19)	0.103
*Categories*							
Q1	1230 (2.5)	1.33 (1.24–1.42)	< 0.001	1.43 (1.34–1.53)	< 0.001	1.15 (1.07–1.23)	< 0.001
Q2–Q4	2700 (1.8)	Ref		Ref		Ref	
*Categories(based on clinical tests below the lower limit)*							
B1	93 (3.8)	2.02 (1.64–2.48)	< 0.001	2.09 (1.70–2.57)	< 0.001	1.42 (1.15–1.74)	0.001
B2	3837 (2.0)	Ref		Ref		Ref	

Participants were divided by sex-specific quartiles (Q1–Q4) of serum creatinine level at baseline: < 71.8, 71.8 ≤ 79.0,79.0 ≤ 86.6 and 86.6–106.0 umol/L for men; < 51.6, 51.6 ≤ 57.0,57.0 ≤ 63.0 and 63.0–97.0 umol/L for women.Participants were divided by sex-specific quartiles (B1–B2)of serum creatinine level at baseline : < 57 and 57.0–106.0 umol/L for men; < 41.0 and 41.0–97.0 umol/L for women.

*Model I*: Adjusted for age and sex

*Model II*: adjusted for all the variables in the model I, plus body mass index, systolic blood pressure, diastolic blood pressure, fasting plasma glucose,total cholesterol,triglyceride, blood urea nitrogen, alanine aminotransferase, drinking status, smoking status and family history of diabetes.

When combining quartiles in further exploratory analysis, a significantly higher risk of new-onset type 2 diabetes was found among participants in quartiles1(adjusted HR, 1.15; 95% CI, 1.07–1.23) compared with those in quartile 2–4 (Table 2).

### Stratified analyses by additional factors

We performed further stratified analyses to assess the association between creatinine (quartile 1 vs quartile2–4) and the risk of new-onset type 2 diabetes in various subgroups (Fig. 2).

**Figure 3:**
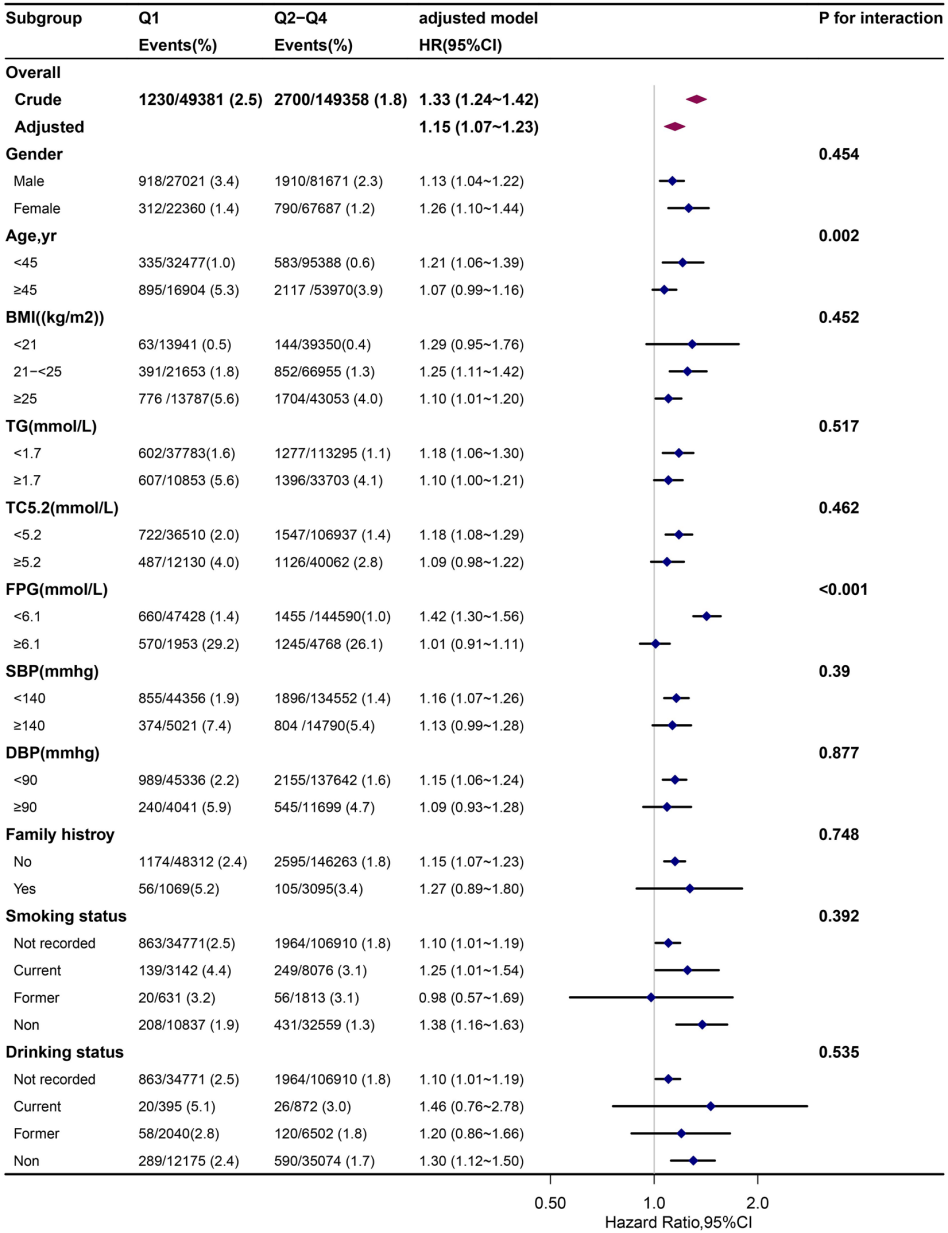
Stratified analysis of the impact of creatinine on new-onset Type 2 Diabetes by other potential effect modifiers. Adjusted for age, sex, body mass index,systolic blood pressure,diastolic blood pressure, fasting plasma glucose, total cholesterol, triglyceride, blood urea nitrogen, alanine aminotransferase, drinking status, smoking status and family history of diabetes, if not be stratified. Boxes denote odds ratios (ORs), lines represent 95% CIs. *BMI* body mass index, *SBP* systolic blood pressure, *DBP* diastolic blood pressure, *FPG* fasting plasma glucose, *TC* total cholesterol, *TG* triglyceride.

None of the variables, including age (< 45 vs. ≥ 45 years), gender(male vs. female), BMI(< 21,21 ≤ 25 vs. ≥ 25 kg/m^2^), TG (< 1.7 vs. ≥ 1.7 mmol/L), TC (< 5.2 vs. ≥ 5.2 mmol/L), FPG (< 6.1 vs. ≥ 6.1 mmol/L), SBP (< 140 vs. ≥ 140 mmHg), DBP (< 90 vs. ≥ 90 mmHg), family history of diabetes (no vs. yes), smoking status (not recorded, current, former vs. non) and drinking status (not recorded, current, former vs. non) at baseline, modified the association between creatinine and new-onset type 2 diabetes. Although the P values for interactions for age and FPG were lower than 0.05, these results may not have significant clinical implications given multiple testing and similar directionality of the associations (Fig. 3).

### Additional exploratory analysis

As an additional exploratory analysis, we used male (n = 108,692) and female (n = 90,047) as a gender cohort. We used Cox proportional risk model assessment for creatinine and new-onset type 2 diabetes, and continued sensitivity subgroup analysis. We get consistent and more stable results (Table S1, Table S2, Table S3, Figure S1 and Figure S2).

## Discussion

To the best of our knowledge, this is a large prospective study of creatinine and diabetes, demonstrating that low serum creatinine is an independent risk factor for new T2DM in Chinese adults with a median follow-up of 3.1 years, compared with high serum creatinine at baseline. Independent of established risk factors for type 2 diabetes, such as BMI, fasting blood glucose (FPG), and a genetic history of diabetes. The relationship was also stable across various stratified analyses.

The only metabolite of phosphocreatine in skeletal muscle is creatinine. Under stable conditions, creatine in skeletal muscle forms creatinine at a relatively constant rate, mainly through non-enzymatic dehydration, and is released into the blood for excretion in urine. Because this blood flesh anhydride and total muscle inside body concern closely, not easy to suffer food effect^8,11^. Studies have shown that muscle mass is closely related to creatinine (correlation coefficient ≥ 0.7)^15,26^.Thus, when renal function is stable and protein intake is normal, creatinine may also be an inexpensive and readily available marker of muscle mass compared to more advanced techniques such as computed tomography, magnetic resonance imaging, or dual-energy X-ray absorption to assess muscle mass^12^.

Low serum creatinine was reported as a risk factor for type 2 diabetes in three small cohort studies of men n = 8570^16^, n = 3313^17^, n = 31343^18^.The study population was single man, and the results limited extrapolation to the general population. Three large Asian population studies (n = 9667^19^, n = 57587^20^and n = 41439^21^participants, also showed an association between serum creatinine levels and the risk of type 2 diabetes in both men and women. In our cohort study, n = 198739, we adjusted for more covariables (established risk factors), including age, sex, BMI, blood pressure, lipids, fasting blood glucose and genetic history of diabetes. We concluded that a more robust low level of creatinine is a risk factor for diabetes.

Current research suggests that low levels of creatinine are a risk factor for diabetes. The range of creatinine in the low-level group currently studied is at baseline: 0.4–0.6^16^, < 0.7^19^, 0.38–0.69^17^, 0.3–0.78 (27–70 umol/L)^20^, < 0.7^18^, < 0.91^21^ mg/dL for men; < 0.50^19^,0.16–0.57 (15–51 umol/L)^20^, < 0.68^21^ mg/dL for women.The low level group in our study was < 0.80(71.8 umol/L) and < 0.58(51.6 umol/L) mg/dL respectively. We had nearly n = 50,000 participants in each group, and after adjusting for variables, the higher risk of diabetes in the lower creatinine group was very stable. We also performed a grouping analysis based on clinical and test thresholds (< 0.80(71.8umol/L) for men and < 0.58(51.6umol/L) mg/dL for women), lower creatinine was associated with a reduced incidence in the population, but adjusted for the corresponding variables, this population was at a higher risk of diabetes.

We also did a detailed stratified analysis. In age stratification at 45 years old, our study shows that low levels of creatinine are associated with a higher risk of diabetes in the young group. Contrary to previous studies, a cohort study of Japanese men^18^ found that low levels of creatinine are associated with a higher risk of diabetes in the elderly, which may be the reason for our large sample size. It also suggests that younger people may be looking at creatinine levels and muscle mass issues while looking at established risk factors for diabetes. The stratification of interest is that baseline fasting glucose is less than 6.1 mmol/ L and low creatinine is associated with a higher risk of diabetes, which needs to be confirmed by further studies.

Our findings have a biological basis based on existing evidence, although the underlying mechanism of the negative association between serum creatinine and type 2 diabetes remains to be described. Studies have shown that skeletal muscle insulin resistance, oxidative stress and inflammation are considered to be the main mechanism leading to the development of diabetes^27,28^. Insulin resistance is a characteristic of type 2 diabetes and may be the primary cause of most type 2 diabetes cases. Compared with obesity and lipid metabolism disorders leading to insulin resistance, skeletal muscle insulin resistance is relatively less studied. Insulin resistance refers to the reduced sensitivity of the target organs of insulin action (mainly liver, muscle and adipose tissue)^28^ to insulin action. Reduced skeletal muscle mass leads to reduced target tissue for insulin action, decreased sensitivity to insulin action, and skeletal muscle insulin resistance, resulting in reduced skeletal muscle glucose uptake and impaired glucose phosphorylation, resulting in impaired skeletal muscle mitochondrial function and metabolic disorders. The accumulation of metabolites (mainly diacylglycerol (DAGs))^28^ and a series of myoinflammatory factors^29,30^ (such as interleukin-6^31^) participate in the impaired autophagy function of cells^32^, and then block insulin signal transduction in skeletal muscle^33^, and interact with each other in this process, resulting in the occurrence and progression of type 2 diabetes. Skeletal muscle undertakes most of the tasks of glucose metabolism, and its decreased ability of insulin stimulated glucose uptake is of great significance for systemic glucose homeostasis^34^. Studies have shown that skeletal muscle has impaired glucose uptake and phosphorylation in the early stage of diabetes^35^, and muscle fiber atrophy in skeletal muscle leads to the occurrence and progression of diabetes^36^. In addition, studies have shown that resistance exercise can improve skeletal muscle insulin resistance^37^, which may be used as an intervention to reduce the occurrence and progression of diabetes. Therefore, we speculate that skeletal muscle insulin resistance may be an important factor in the increased risk of new-onset diabetes. In general, future studies need to further study the potential mechanisms of serum creatinine and muscle mass on the occurrence of diabetes.

Our study also has some limitations. Firstly, although a series of covariables were adjusted in the regression model, residual confounding effects from unmeasured or unknown factors could not be excluded. Secondly, our study was a cohort with a large sample size, which could overcome some confounding effects. Second, our current study was conducted among Chinese adults, and further investigation is needed to determine whether the observed results can be extrapolated to other populations. Third, we do not have detailed information on diet, but our participants are generally healthy urban adults who follow the Traditional Chinese diet for three meals a day. Therefore, there is little difference between individuals^38^, and it is difficult to achieve complete consistency in diet in a large cohort study of the general population. Fourthly, we used the diagnostic criteria for diabetes without oral glucose tolerance test. Some studies have shown that the incidence of diabetes may be lower if fasting glucose is used to diagnose diabetes, however, it is noteworthy that low incidence of diabetes resulting from such errors would bias toward the null, thus, resulting in an underestimation of the association between creatinine and type 2 diabetes. Overall, our results need to be confirmed in future studies.

In conclusion, low serum creatinine is independently correlated with increased risk of incident diabetes in Chinese adults. It was also stable in various subgroups stratified.

## Supplementary Information

Supplementary Information.

## Data Availability

The data are available at https://datadryad.org/stash/dataset/doi:10.5061%2Fdryad.ft8750v which allows researchers to freely download the original data.
