# Supplementary material for: Association between serum creatinine and type 2 diabetes in the Chinese population: a retrospective cohort study
Source: Sci Rep. 2023 Apr 26;13:6806. doi: 10.1038/s41598-023-33878-6 (PMC10133309; doi:10.1038/s41598-023-33878-6)
Supplement: Supplementary file 1 — Supplementary Information. [file 41598_2023_33878_MOESM1_ESM.pdf]

# *Supplementary Material*

**Table S1** Baseline characteristics of male patients

| Variables | Total | Creatinine quartile(umol/L) | | | | P-value |
|---|---|---|---|---|---|---|
| | | Q1(<71.8) | Q2(71.8-<79.0) | Q3(79.0-<86.6) | Q4(86.6-106.0) | |
| No of subjects | 108692 | 27021 | 27092 | 2738 | 27198 | |
| Age，years | 42.3 ± 12.8 | 42.5 ± 12.7 | 41.8 ± 12.5 | 41.9 ± 12.7 | 42.7 ± 13.5 | < 0.001 |
| BMI(kg/m2 ) | 24.2 ± 3.2 | 24.1 ± 3.4 | 24.1 ± 3.3 | 24.2 ± 3.2 | 24.4 ± 3.1 | < 0.001 |
| SBP(mmHg) | 122.7 ± 15.4 | 123.4 ± 15.6 | 122.4 ± 15.2 | 122.3 ± 15.2 | 122.7 ± 15.6 | < 0.001 |
| DBP(mmHg) | 76.6 ± 10.6 | 76.9 ± 10.7 | 76.5 ± 10.5 | 76.4 ± 10.4 | 76.5 ± 10.7 | < 0.001 |
| FPG(mmol/L) | 5.0 ± 0.6 | 5.0 ± 0.6 | 5.0 ± 0.6 | 4.9 ± 0.6 | 5.0 ± 0.6 | < 0.001 |
| TC(mmol/L) | 4.7 ± 0.9 | 4.7 ± 0.9 | 4.7 ± 0.9 | 4.7 ± 0.9 | 4.8 ± 0.9 | < 0.001 |
| TG(mmol/L) | 1.6 ± 1.2 | 1.6 ± 1.4 | 1.6 ± 1.1 | 1.6 ± 1.1 | 1.6 ± 1.1 | < 0.001 |
| HDL-C(mmol/L) | 2.8 ± 0.7 | 2.8 ± 0.7 | 2.8 ± 0.7 | 2.8 ± 0.7 | 2.8 ± 0.7 | < 0.001 |
| LDL-C(mmol/L) | 1.3 ± 0.3 | 1.3 ± 0.3 | 1.3 ± 0.3 | 1.3 ± 0.3 | 1.3 ± 0.3 | 0.011 |
| ALT(IU/L) | 23.0 (16.5, 34.2) | 23.7 (16.9, 35.5) | 23.1 (16.5, 34.8) | 23.0 (16.3, 34.0) | 22.7 (16.3, 33.1) | < 0.001 |
| AST(IU/L) | 23.5 (20.0, 28.7) | 23.8 (20.0, 29.0) | 23.7 (20.0, 28.8) | 23.3 (20.0, 28.0) | 23.3 (20.0, 28.4) | < 0.001 |
| Bun(mmol/L) | 4.9 ± 1.2 | 4.7 ± 1.1 | 4.8 ± 1.1 | 5.0 ± 1.1 | 5.2 ± 1.2 | < 0.001 |
| Family history of diabetes | 1554 (1.4) | 346 (1.3) | 406 (1.5) | 384 (1.4) | 418 (1.5) | 0.056 |
| Drinking status (%) | | | | | | < 0.001 |
| Not recorded | 71772 (66.0) | 17827 (66) | 17565 (64.8) | 17913 (65.4) | 18467 (67.9) | |
| Current | 11190 (10.3) | 3135 (11.6) | 2928 (10.8) | 2737 (10) | 2390 (8.8) | |
| Former | 2422 (2.2) | 624 (2.3) | 642 (2.4) | 580 (2.1) | 576 (2.1) | |
| Non | 23308 (21.4) | 5435 (20.1) | 5957 (22) | 6151 (22.5) | 5765 (21.2) | |
| Smoking status (%) | | | | | | < 0.001 |
| Not recorded | 71772 (66.0) | 17827 (66) | 17565 (64.8) | 17913 (65.4) | 18467 (67.9) | |
| Current | 1241 (1.1) | 387 (1.4) | 305 (1.1) | 317 (1.2) | 232 (0.9) | |
| Former | 8018 (7.4) | 1892 (7) | 2033 (7.5) | 2077 (7.6) | 2016 (7.4) | |
| Non | 27661 (25.4) | 6915 (25.6) | 7189 (26.5) | 7074 (25.8) | 6483 (23.8) | |

**Abbreviations:** BMI,body mass index;SBP,systolic blood pressure;DBP,diastolic blood pressure;FPG,fasting plasma glucose;TC,total cholesterol;TG,triglyceride;LDL-C,low-density lipid cholesterol;HDL-C, high-density lipoprotein cholesterol;BUN,blood urea nitrogen;ALT,alanine aminotransferase;AST,aspartate aminotransferase.

**Table S2** Baseline characteristics of female patients

| Variables | Total | Creatinine quartile(umol/L) | | | | P-value |
|---|---|---|---|---|---|---|
| | | Q1(<51.6) | Q2(51.6 -<57.0) | Q3(57.0-<63.0) | Q4(63.0-97.0) | |
| No of subjects | 90047 | 22360 | 21761 | 22668 | 23258 | |
| Age，years | 42.0 ± 12.4 | 40.2 ± 10.9 | 41.0 ± 11.6 | 42.0 ± 12.2 | 44.7 ± 14.1 | < 0.001 |
| BMI(kg/m2 ) | 22.1 ± 3.1 | 22.0 ± 3.1 | 22.0 ± 3.1 | 22.0 ± 3.0 | 22.3 ± 3.1 | < 0.001 |
| SBP(mmHg) | 114.4 ± 16.3 | 114.1 ± 15.8 | 113.6 ± 15.7 | 114.0 ± 16.1 | 116.0 ± 17.4 | < 0.001 |
| DBP(mmHg) | 71.2 ± 10.3 | 71.0 ± 10.2 | 70.9 ± 10.1 | 71.1 ± 10.2 | 71.6 ± 10.5 | < 0.001 |
| FPG(mmol/L) | 4.8 ± 0.6 | 4.9 ± 0.6 | 4.8 ± 0.6 | 4.8 ± 0.6 | 4.9 ± 0.6 | < 0.001 |
| TC(mmol/L) | 4.7 ± 0.9 | 4.6 ± 0.9 | 4.7 ± 0.9 | 4.7 ± 0.9 | 4.8 ± 0.9 | < 0.001 |
| TG(mmol/L) | 1.1 ± 0.7 | 1.0 ± 0.8 | 1.0 ± 0.7 | 1.0 ± 0.7 | 1.1 ± 0.7 | < 0.001 |
| HDL-C(mmol/L) | 1.5 ± 0.3 | 1.5 ± 0.3 | 1.5 ± 0.3 | 1.5 ± 0.3 | 1.5 ± 0.3 | < 0.001 |
| LDL-C(mmol/L) | 2.7 ± 0.7 | 2.7 ± 0.7 | 2.7 ± 0.7 | 2.7 ± 0.7 | 2.8 ± 0.7 | < 0.001 |
| ALT(IU/L) | 13.9 (10.9, 19.0) | 14.0 (10.9, 19.4) | 13.6 (10.7, 18.5) | 13.7 (10.8, 18.6) | 14.0 (11.0, 18.8) | < 0.001 |
| AST(IU/L) | 20.0 (17.1, 24.0) | 20.0 (17.0, 23.6) | 20.0 (17.0, 23.6) | 20.0 (17.0, 23.7) | 20.4 (17.5, 24.3) | < 0.001 |
| Bun(mmol/L) | 4.3 ± 1.1 | 4.1 ± 1.0 | 4.2 ± 1.1 | 4.4 ± 1.1 | 4.6 ± 1.1 | < 0.001 |
| Family history of diabetes | 2610 (2.9) | 723 (3.2) | 640 (2.9) | 627 (2.8) | 620 (2.7) | 0.002 |
| Drinking status (%) | | | | | | < 0.001 |
| Not recorded | 69909 (77.6) | 16944 (75.8) | 16621 (76.4) | 17670 (78) | 18674 (80.3) | |
| Current | 26 (0.0) | 8 (0) | 8 (0) | 5 (0) | 5 (0) | |
| Former | 524 (0.6) | 148 (0.7) | 128 (0.6) | 127 (0.6) | 121 (0.5) | |
| Non | 19588 (21.8) | 5260 (23.5) | 5004 (23) | 4866 (21.5) | 4458 (19.2) | |
| Smoking status (%) | | | | | | < 0.001 |
| Not recorded | 69909 (77.6) | 16944 (75.8) | 16621 (76.4) | 17670 (78) | 18674 (80.3) | |
| Current | 28 (0.0) | 7 (0) | 4 (0) | 9 (0) | 8 (0) | |
| Former | 22 (0.0) | 7 (0) | 8 (0) | 4 (0) | 3 (0) | |
| Non | 20088 (22.3) | 5402 (24.2) | 5128 (23.6) | 4985 (22) | 4573 (19.7) | |

Abbreviations: BMI,body mass index;SBP,systolic blood pressure;DBP,diastolic blood pressure;FPG,fasting plasma glucose;TC,total cholesterol;TG,triglyceride;LDL-C,low-density lipid cholesterol;HDL-C, high-density lipoprotein cholesterol;BUN,blood urea nitrogen;ALT,alanine aminotransferase;AST,aspartate aminotransferase.

**Table S3** Association between creatinine and type 2 diabetes

| Creatinine | N | n.event(%) | Crude Model | | Adjusted Model I | | Adjusted Model II | |
|---|---|---|---|---|---|---|---|---|
| | | | HR (95%CI） | *P* value | HR (95%CI） | *P* value | HR (95%CI） | *P* value |
| **Female Creatinine Quartiles** | | | | | | | | |
| Q1(<51.6) | 22360 | 312 (1.4) | 1.36 (1.15~1.61) | | 1.63 (1.38~1.93) | <0.001 | 1.33 (1.12~1.58) | 0.001 |
| Q2(51.6-<57) | 21761 | 227 (1) | 1 .00(0.83~1.2) | 0.989 | 1.13 (0.94~1.35) | 0.20 | 1.04 (0.86~1.25) | 0.688 |
| Q3(57-<63) | 22668 | 241 (1.1) | Ref | | Ref | | Ref | |
| Q4(63-97) | 23258 | 322 (1.4) | 1.40 (1.19~1.66) | <0.001 | 0.97 (0.82~1.15) | 0.749 | 1.09 (0.92~1.29) | 0.306 |
| Trend.test | 90047 | 1102 (1.2) | | <0.001 | | <0.001 | | <0.001 |
| **Female Categories** | | | | | | | | |
| Q1(<51.6) | 22360 | 312 (1.4) | 1.20(1.05~1.37) | 0.007 | 1.59 (1.39~1.82) | <0.001 | 1.27 (1.11~1.46) | 0.001 |

| | | | | | | | | |
|---|---|---|---|---|---|---|---|---|
| Q2-Q4(51.6-97) | 67687 | 790 (1.2) | Ref | | Ref | | Ref | |
| **Male Creatinine Quartiles** | | | | | | | | |
| Q1(<71.8) | 27021 | 918 (3.4) | 1.48 (1.34~1.64) | <0.001 | 1.47 (1.32~1.63) | <0.001 | 1.21(1.09~1.34) | <0.001 |
| Q2(71.8-<79) | 27092 | 713 (2.6) | 1.15 (1.03~1.28) | 0.012 | 1.17 (1.05~1.31) | 0.004 | 1.12 (1~1.25) | 0.041 |
| Q3(79-<86.6) | 27381 | 596 (2.2) | Ref | | Ref | | Ref | |
| Q4(86.6-106) | 27198 | 601 (2.2) | 1.12 (1.00~1.26) | 0.043 | 0.99 (0.88~1.11) | 0.846 | 1.08(0.96~1.21) | 0.193 |
| Trend.test | 108692 | 2828 (2.6) | | <0.001 | | <0.001 | | 0.005 |
| **Male Categories** | | | | | | | | |
| Q1(<71.8) | 27021 | 918 (3.4) | 1.36 (1.26~1.47) | <0.001 | 1.39 (1.28~1.50) | <0.001 | 1.13 (1.04~1.22) | 0.003 |
| Q2-Q4(71.8-106) | 81671 | 1910 (2.3) | Ref | | Ref | | Ref | |

**Notes:** Participants were divided by sex-specific quartiles (Q1-Q4) of serum creatinine level at baseline:<71.8, 71.8-<79.0,79.0-<86.6 and 86.6-106.0 umol/L for men;<51.6, 51.6-<57.0,57.0-<63.0 and 63.0-97.0 umol/L for women.

**Model I:** Adjusted for age body mass index, systolic blood pressure,and diastolic blood pressure.

**Model II**: adjusted for all the variables in the model I, plus fasting plasma glucose,total cholesterol,triglyceride,blood urea nitrogen,alanine aminotransferase,drinking status,smoking status and family history of diabetes.

| Subgroup | Q1 Events(%) | Q2−4 Events(%) | adjusted model HR(95%CI) | P for interaction |
|---|---|---|---|---|
| **Overall** | | | | |
| **Crude** | **1910/81671 (2.3)** | **918/27021 (3.4)** | **1.36 (1.26~1.47)** | |
| **Adjusted** | | | **1.13 (1.04~1.22)** | |
| **Age,yr** | | | | **0.004** |
| <45 | 465/52746 (0.9) | 257/16759(1.5) | 1.22 (1.05~1.43) | |
| ≥45 | 1445 /28907(5.0) | 661/10262 (6.4) | 1.05 (0.96~1.16) | |
| **BMI((kg/m2))** | | | | **0.366** |
| <21 | 49/12487 (0.4) | 35/4859(0.7) | 1.70(1.08~2.67) | |
| 21−<25 | 532/36215 (1.5) | 257/11701 (2.2) | 1.22 (1.04~1.41) | |
| ≥25 | 1329/32969 (4.0) | 626 /10461(6.0) | 1.11 (1.01~1.22) | |
| **TG(mmol/L)** | | | | **0.738** |
| <1.7 | 828/54708 (1.5) | 418/18237 (2.3) | 1.14 (1.01~1.29) | |
| ≥1.7 | 1057/25566 (4.1) | 481/8344 (5.8) | 1.10(0.99~1.23) | |
| **TC5.2(mmol/L)** | | | | **0.643** |
| <5.2 | 1170/58077 (2.0) | 555/19403 (2.9) | 1.16 (1.04~1.28) | |
| ≥5.2 | 715/22198 (3.2) | 344/7178 (4.8) | 1.07 (0.94~1.22) | |
| **FPG(mmol/L)** | | | | **<0.001** |
| <6.1 | 1002 /78342(1.3) | 477/25617 (1.9) | 1.38 (1.24~1.55) | |
| ≥6.1 | 908/3329 (27.3) | 441/1404 (31.4) | 1.01 (0.90~1.13) | |
| **SBP(mmhg)** | | | | **0.476** |
| <130 | 943/57248 (1.6) | 449 /18293(2.5) | 1.13 (1.01~1.27) | |
| ≥130 | 967/24415 (4.0) | 469/8726 (5.4) | 1.11 (0.99~1.24) | |
| **DBP(mmhg)** | | | | **0.624** |
| <80 | 875/52476 (1.7) | 413/16804 (2.5) | 1.07 (0.95~1.21) | |
| ≥80 | 1035/29187 (3.5) | 505/10215 (4.9) | 1.15 (1.03~1.29) | |
| **Smoking status** | | | | **0.958** |
| Not recorded | 1324 /53945(2.5) | 640/17827 (3.6) | 1.12 (1.02~1.23) | |
| Non | 249/8055(3.1) | 138 /3135(4.4) | 1.27 (1.03~1.57) | |
| Former | 54/1798 (3.0) | 20/624 (3.2) | 1.04 (0.61~1.8) | |
| Current | 283 /17873(1.6) | 120/5435 (2.2) | 1.13 (0.90~1.41) | |
| **Drinking status** | | | | **0.578** |
| Not recorded | 1324/53945 (2.5) | 640 /17827(3.6) | 1.12 (1.02~1.23) | |
| Non | 26/854 (3.0) | 20 /387(5.2) | 1.47 (0.78~2.78) | |
| Former | 119/6126 (1.9) | 54 /1892(2.9) | 1.13 (0.80~1.57) | |
| Current | 441 /20746(2.1) | 204 /6915(3.0) | 1.16 (0.98~1.38) | |
| **Family histroy** | | | | **0.071** |
| No | 1841/80463 (2.3) | 891/26675 (3.3) | 1.14 (1.05~1.24) | |
| Yes | 69/1208 (5.7) | 27/346 (7.8) | 0.89 (0.54~1.48) | |

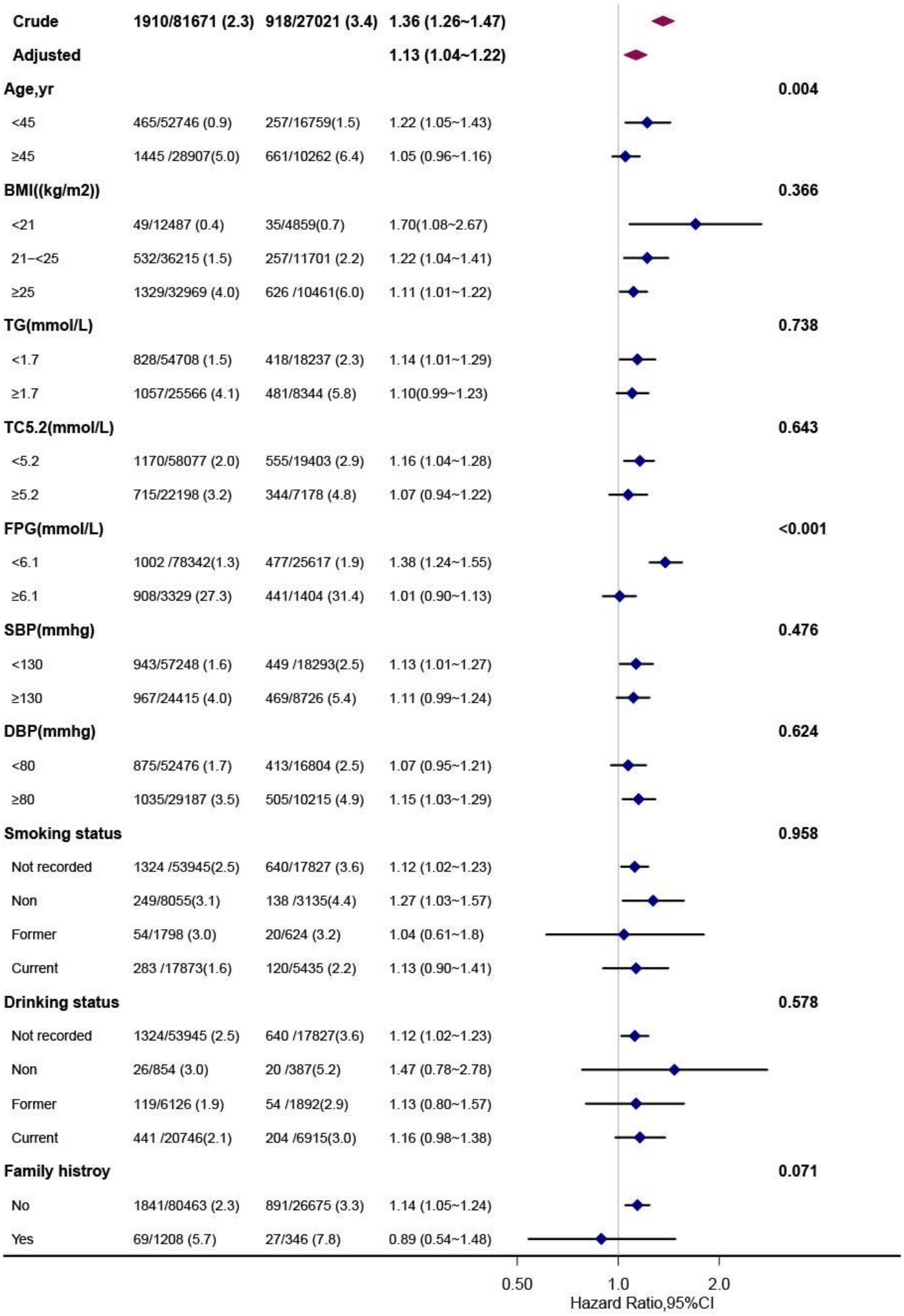


**Figure S1** Stratified analysis of the impact of creatinine on new-onset Type 2 Diabetes by

other potential effect modifiers for male**.** Adjusted for age, body mass index,systolic blood pressure,diastolic blood pressure,fasting plasma glucose,total cholesterol,triglyceride,blood urea nitrogen,alanine aminotransferase,drinking status,smoking status and family history of diabetes, if not be stratified. Boxes denote odds ratios (ORs), lines represent 95% CIs.

**Abbreviations:**BMI,body mass index;SBP,systolic blood pressure;DBP,diastolic blood pressure;FPG,fasting plasma glucose;TC,total cholesterol;TG,triglyceride.

| Subgroup | Q1 Events(%) | Q2−4 Events(%) | adjusted model HR(95%CI) | P for interaction |
|---|---|---|---|---|
| **Overall** | | | | |
| **Crude** | **790/67687 (1.2)** | **312/22360 (1.4)** | **1.20 (1.05~1.37)** | |
| **Adjusted** | | | **1.27 (1.11~1.45)** | |
| **Age,yr** | | | | **0.124** |
| <45 | 118/42624 (0.3) | 78 /15718(0.5) | 1.23 (0.92~1.65) | |
| ≥45 | 672/25063 (2.7) | 234/6642 (3.5) | 1.13 (0.97~1.32) | |
| **BMI(kg/m2)** | | | | **0.331** |
| <21 | 95/26863 (0.4) | 28/9082 (0.3) | 1.01 (0.66~1.55) | |
| 21−<25 | 290/29758 (1.0) | 121 /9690(1.2) | 1.38 (1.11~1.72) | |
| ≥25 | 405/11066 (3.7) | 163 /3588(4.5) | 1.14 (0.94~1.38) | |
| **TG(mmol/L)** | | | | **0.602** |
| <1.7 | 449/58587 (0.8) | 184/19546 (0.9) | 1.32 (1.10~1.57) | |
| ≥1.7 | 339/8137 (4.2) | 126 /2509(5.0) | 1.14 (0.91~1.41) | |
| **TC(mmol/L)** | | | | **0.752** |
| <5.2 | 377/48860 (0.8) | 167/17107 (1.0) | 1.28 (1.07~1.55) | |
| ≥5.2 | 411/17864 (2.3) | 143/4952 (2.9) | 1.21 (0.99~1.48) | |
| **FPG(mmol/L)** | | | | **0.211** |
| <6.1 | 453/66248 (0.7) | 183 /21811(0.8) | 1.61 (1.35~1.92) | |
| ≥6.1 | 337/1439 (23.4) | 129/549 (23.5) | 1.01(0.81~1.23) | |
| **SBP(mmhg)** | | | | **0.567** |
| <130 | 350 /56219(0.6) | 140/18876 (0.7) | 1.20 (0.98~1.47) | |
| ≥130 | 440/11460 (3.8) | 171 /3482(4.9) | 1.28 (1.06~1.54) | |
| **DBP(mmhg)** | | | | **0.563** |
| <80 | 460 /54148(0.8) | 169/17967 (0.9) | 1.25 (1.04~1.50) | |
| ≥80 | 330/13530 (2.4) | 142/4391 (3.2) | 1.26 (1.02~1.55) | |
| **Family histroy** | | | | **0.101** |
| No | 754/65800 (1.1) | 283/21637 (1.3) | 1.25 (1.08~1.44) | |
| Yes | 36/1887 (1.9) | 29/723 (4.0) | 1.87 (1.12~3.13) | |

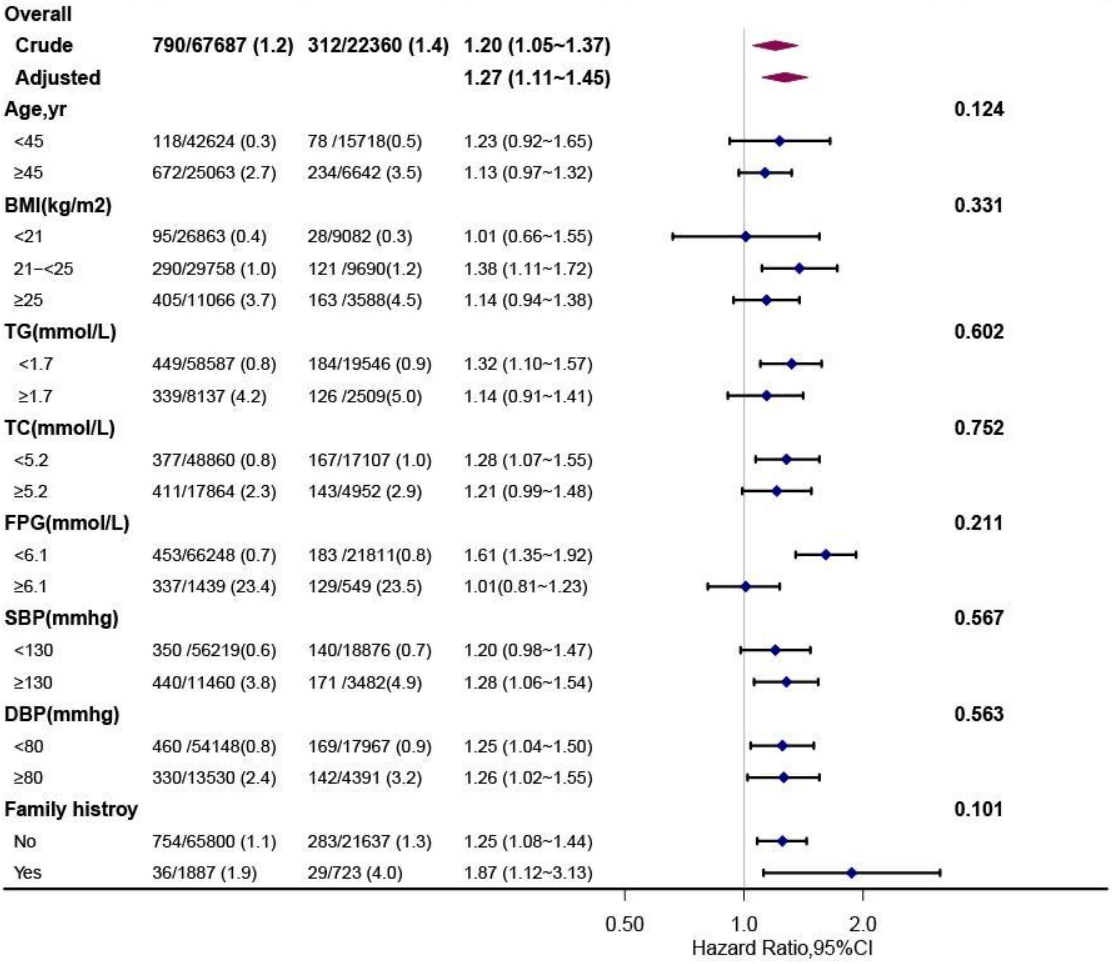


**Figure S2** Stratified analysis of the impact of creatinine on new-onset Type 2 Diabetes by other potential effect modifiers for female**.** Adjusted for age, body mass index,systolic blood pressure,diastolic blood pressure,fasting plasma glucose,total cholesterol,triglyceride,blood urea nitrogen,alanine aminotransferase,drinking status,smoking status and family history of diabetes, if not be stratified. Boxes denote odds ratios (ORs), lines represent 95% CIs.

**Abbreviations:**BMI,body mass index;SBP,systolic blood pressure;DBP,diastolic blood pressure;FPG,fasting plasma glucose;TC,total cholesterol;TG,triglyceride.
